# Alzheimer's disease‐associated CD83(+) microglia are linked with increased immunoglobulin G4 and human cytomegalovirus in the gut, vagal nerve, and brain

**DOI:** 10.1002/alz.14401

**Published:** 2024-12-19

**Authors:** Benjamin P. Readhead, Diego F. Mastroeni, Qi Wang, Maria A. Sierra, Camila de Ávila, Tajudeen O. Jimoh, Jean‐Vianney Haure‐Mirande, Kristina E. Atanasoff, Jennifer Nolz, Crystal Suazo, Nathaniel J. Barton, Adrian R. Orszulak, Samantha M. Chigas, Khanh Tran, Anne Mirza, Krista Ryon, Jacqueline Proszynski, Deena Najjar, Joel T. Dudley, Sean T. H. Liu, Sam Gandy, Michelle E. Ehrlich, Eric Alsop, Jerry Antone, Rebecca Reiman, Cory Funk, Rebecca L. Best, Michael Jhatro, Kathy Kamath, John Shon, Timothy F. Kowalik, David A. Bennett, Winnie S. Liang, Geidy E. Serrano, Thomas G. Beach, Kendall Van Keuren‐Jensen, Christopher E. Mason, Yingleong Chan, Elaine T. Lim, Domenico Tortorella, Eric M. Reiman

**Affiliations:** ^1^ ASU‐Banner Neurodegenerative Disease Research Center Arizona State University Tempe Arizona USA; ^2^ Department of Physiology and Biophysics Weill Cornell Medicine New York New York USA; ^3^ Department of Microbiology Icahn School of Medicine at Mount Sinai New York New York USA; ^4^ Department of Neurology Icahn School of Medicine at Mount Sinai New York New York USA; ^5^ Department of Neurology University of Massachusetts Chan Medical School Worcester Massachusetts USA; ^6^ Department of Genomics and Computational Biology University of Massachusetts Chan Medical School Worcester Massachusetts USA; ^7^ Department of Microbiology and Physiological Systems University of Massachusetts Chan Medical School Worcester Massachusetts USA; ^8^ Department of Physiology and Biophysics Weill Cornell Medicine New York New York USA; ^9^ Division of Infectious Diseases Icahn School of Medicine at Mount Sinai New York New York USA; ^10^ Division of Neurogenomics The Translational Genomics Research Institute Phoenix Arizona USA; ^11^ Institute for Systems Biology Seattle Washington USA; ^12^ Serimmune, Inc Goleta California USA; ^13^ Rush Alzheimer's Disease Center Rush University Medical Center Chicago Illinois USA; ^14^ Civin Laboratory for Neuropathology Banner Sun Health Research Institute Sun City Arizona USA; ^15^ Department of Medicine Division of Innate Immunity University of Massachusetts Chan Medical School Worcester Massachusetts USA; ^16^ Banner Alzheimer's Institute Phoenix Arizona USA

**Keywords:** Alzheimer's disease, antibody epitope repertoire analysis, CD83(+) microglia, cerebrospinal fluid, human cytomegalovirus, immunoglobulin G4, immunohistochemistry, prefrontal cortex, superior frontal gyrus, transverse colon, vagus nerve, viral infection

## Abstract

**INTRODUCTION:**

While there may be microbial contributions to Alzheimer's disease (AD), findings have been inconclusive. We recently reported an AD‐associated CD83(+) microglia subtype associated with increased immunoglobulin G4 (IgG4) in the transverse colon (TC).

**METHODS:**

We used immunohistochemistry (IHC), IgG4 repertoire profiling, and brain organoid experiments to explore this association.

**RESULTS:**

CD83(+) microglia in the superior frontal gyrus (SFG) are associated with elevated IgG4 and human cytomegalovirus (HCMV) in the TC, anti‐HCMV IgG4 in cerebrospinal fluid, and both HCMV and IgG4 in the SFG and vagal nerve. This association was replicated in an independent AD cohort. HCMV‐infected cerebral organoids showed accelerated AD pathophysiological features (Aβ42 and pTau‐212) and neuronal death.

**DISCUSSION:**

Findings indicate complex, cross‐tissue interactions between HCMV and the adaptive immune response associated with CD83(+) microglia in persons with AD. This may indicate an opportunity for antiviral therapy in persons with AD and biomarker evidence of HCMV, IgG4, or CD83(+) microglia.

**Highlights:**

- Cross‐tissue interaction between HCMV and the adaptive immune response in a subset of persons with AD.
- Presence of CD83(+) microglial associated with IgG4 and HCMV in the gut.
- CD83(+) microglia are also associated presence of HCMV and IgG4 in the cortex and vagal nerve.
- Replication of key association in an independent cohort of AD subjects.
- HCMV infection of cerebral organoids accelerates the production of AD neuropathological features.

## 1 BACKGROUND

The emergence of single nucleus RNA sequencing (snRNAseq) studies of Alzheimer's disease (AD) and aging‐affected brain tissue has demonstrated the powerful opportunity for cell transcriptomics to illuminate and resolve disease mechanisms in a novel and informative manner that cannot be accomplished by bulk tissue profiling alone.^1^, ^2^, ^3^ We recently reported findings^4^ built upon snRNAseq profiles from 481,840 nuclei collected from *post mortem* superior frontal gyrus (SFG) cortical tissue samples from 101 (AD *n* = 66, aged controls *n* = 35) exceptionally well‐characterized, aged subjects from the Arizona Study of Aging and Neurodegenerative Disorders (AZSAND)/Brain and Body Donation Program (BBDP).^5^ We identified a differentially abundant AD‐associated CD83(+) microglial subtype, detected in 47% of AD subjects and 25% of clinically and neuropathologically unaffected controls. Although we refer to this microglia subpopulation as CD83(+) for brevity, these cells are characterized by additional marker genes beyond CD83.^4^ Among subjects with AD, the presence of CD83(+) microglia was not associated with apolipoprotein E4 (APOE4) status, common disease comorbidities, demographic factors, or terminal pneumonia, though was associated with increased multiregional neuritic plaque density and neurofibrillary tangle burden. Network analyses implicated several known microglial genes, including *SPP1*, *TREM2*, and *APOE*, though the most influential network driver was long noncoding RNA *AC131944.1*, an encoded antisense transcript to *SPP1*. Pathway enrichment relative to other microglia highlighted biological themes of senescence, complement activation, ferritin and iron processing, lipid processing, and antibody response. Given that CD83 is a marker of mature dendritic cells, with complex, bidirectional interactions with diverse pathogens^6^, ^7^, ^8^ and its role in activated microglia during neuroinflammation,^9^ we hypothesized that CD83(+) AD subjects may differ from CD83(−) AD subjects on the basis of a microbial or immunological perturbation. Mass spectrometry proteomics data from frozen transverse colon (TC) samples collected from a subset of 26 subjects with SFG snRNAseq revealed the most differentially abundant protein in subjects with CD83(+) microglia was immunoglobulin heavy constant gamma 4 (IGHG4) which forms the constant region of the immunoglobulin IgG4 antibody heavy chain. This observation was suggestive of increased IgG4 tissue response in the TC of AD subjects with CD83(+) microglia and more broadly, consistent with a potential microbial interaction between components of the gut microbiome and the presence of CD83(+) microglia. The potential role microbes on the risk of developing, or accelerating the progress of, AD was first proposed by Alois Alzheimer^10^ and Oskar Fischer^11^ well over a century ago. Although there may be microbial contributions to AD,^12^, ^13^, ^14^, ^15^, ^16^, ^17^, ^18^, ^19^, ^20^, ^21^, ^22^ observations have been complex and at times discordant, with no single pathogen consistently linked to the disease. In this current study, we extend our investigations of the potential etiology and clinicopathological relevance of CD83(+) microglia in the context of AD, integrating molecular profiles from additional anatomical sites collected from the same subjects that had previously undergone SFG snRNAseq.

## 2 METHODS

### 2.1 Cohort design and sample selection

Samples used for analyses of the “Banner cohort” were selected from within a cohort of 101 aged subjects (AD *n* = 66, Aged Controls *n* = 35) that were recently reported within,^4^ and which have undergone extensive clinical and *post mortem* neuropathological characterizations through the AZSAND/BBDP.^5^ Due to the snRNA‐seq performed on SFG cortical tissue by,^4^ we also possessed classifications for the presence of CD83(+) microglia for each subject. Considerations of (a) presence of CD83(+) microglia, (b) AD/Aged control status, and (c) tissue availability were the key heuristics for further sample selection of multi‐tissue sites (SFG, TC, vagus nerve). This resulted in partly overlapping sets of subjects that were selected for IgG4 and HCMV immunohistochemistry (IHC) for each tissue (Table S1).

For validation of the association between CD83(+) microglia and HCMV within cortical tissue (Figure S1), we obtained prefrontal cortex (PFC) tissue samples from 30 AD subjects included in recent snRNA‐seq publications by Fujita et al.^23^ and Mathys et al.^24^ from participants of the Religious Orders Study and Rush Memory and Aging Project (ROSMAP) cohort.^25^ As described in,^4^ we reprocessed available snRNA‐seq data to generate classifications for the presence of CD83(+) microglia for each subject, and then requested frozen sections from PFC tissue for 30 AD subjects (CD83(+) AD *n* = 15, CD83(−) AD *n* = 15). Three samples were excluded due to frozen artifact, leaving a total of 27 AD subjects (CD83(+) AD *n* = 13, CD83(−) AD *n* = 13) for inclusion in analyses shown in Figure S1.

**RESEARCH IN CONTEXT**

1. **Systematic review**: In a single nucleus RNA sequencing study, we recently found a CD83(+) microglial subtype in the superior frontal gyrus (SFG) of 47% of brain donors with Alzheimer's disease (AD) versus 25% of unaffected controls. Here we report that the presence of CD83(+) microglial in the SFG is significantly associated with elevated immunoglobulin IgG4 and human cytomegalovirus (HCMV) in the transverse colon (TC), increased anti‐HCMV IgG4 abundance in the cerebrospinal fluid, and the presence of both HCMV and Immunoglobulin G4 (IgG4) in the SFG and vagal nerve.
2. **Interpretation**: Our results indicate a complex, cross‐tissue interaction between HCMV and the host adaptive immune response associated with CD83(+) microglia in persons with AD.
3. **Future directions**: Histochemical studies are consistent with an active HCMV infection, suggesting an opportunity for the evaluation of antiviral therapy in persons with AD and biomarker evidence of HCMV, IgG4, or CD83(+) microglia.

### 2.2 Banner cohort sample collection and characterizations

Subjects within the Banner cohort were all volunteers in the AZSAND, a longitudinal clinicopathological study of aging, cognition, and movement in the elderly since 1996 in Sun City, Arizona. Autopsies are performed by the Banner Sun Health Research Institute Brain and Body Donation Program^5^ (BBDP; www.brainandbodydonationprogram.org). All subjects sign Institutional Review Board‐approved informed consents allowing both clinical assessments during life and several options for brain and/or bodily organ donation after death. Most subjects are clinically characterized with annual standardized test batteries consisting of general neurological, cognitive, and movement disorders components, including the Mini‐Mental State Examination (MMSE).

The complete neuropathological examination was performed using standard AZSAND methods.^5^ The neuropathological examination was performed in a standardized manner and consisted of gross and microscopic observations, the latter including assessment of frontal, parietal, temporal, and occipital lobes, all major diencephalic nuclei and major subdivisions of the brainstem, cerebellum, and spinal cord (the lattermost only for those with whole‐body autopsy). Detailed clinical data, *post mortem* neuropathological data, and demographics of the cohort are reported in Table S1.

### 2.3 ROSMAP cohort sample collection and characterizations

The ROSMAP are prospective cohort studies of aging and dementia.^25^ Participants without known dementia agree to annual clinical evaluation and brain donation.^25^ Details of the clinical and pathologic methods have been previously reported.^26^, ^27^, ^28^, ^29^, ^30^ Both studies were approved by an Institutional Review Board of Rush University Medical Center. All participants signed informed and repository consents, and an Anatomic Gift Act. Pathologic methods and APOE genotyping have been previously reported.^27^, ^31^, ^32^ Clinical, *post mortem* neuropathological data and demographics of the cohort used for immunohistochemical (IHC) studies are reported in Table S1.

### 2.4 IHC

IHC studies of subjects within the Banner cohort were completed on a total of 34 human SFG samples (BA8) (AD *n* = 21, Aged controls *n* = 13). Additionally, 25 TC samples (AD *n* = 13, Aged control *n* = 12) and 8 vagal nerve samples (AD *n* = 6, Aged control *n* = 2) were included in the study. IHC studies of subjects within the ROSMAP cohort were completed on a total of 27 human PFC samples from subjects with AD. The samples were carefully selected to ensure matching for critical factors such as *post mortem* interval (PMI), age, and sex, as well as other relevant covariates (detailed information can be found in Table S1).

For the free‐floating sections, a DAB (3, 3′‐diaminobenzidine) staining method was employed, following a previously described protocol (in ref. [^33^, ^34^, ^35^, ^36^]). Briefly, 40 *µ*m free‐floating sections were initially blocked in H2O2 for 30 min, followed by blocking in 3% bovine serum albumin (BSA) for 1 h. Subsequently, the sections were incubated with primary antibodies Anti‐Cytomegalovirus Antibody, late Antigen, clone 1G5.2, 1:2000, Sigma Aldrich, MAB8127; Anti‐HCMV IE1/2, early Antigen, 1:2500 (Gift from Tortorella); Anti‐IgG4 antibody (EP4420), 1:2000 ABCAM (ab109493) overnight at 4°C. After thorough washing with phosphate‐buffered saline containing 0.01% Triton X‐100 (PBST), species‐specific biotinylated secondary antibodies (Vector, diluted 1:1000,) were applied for 2 h at room temperature. The sections were then washed with PBST, incubated in an avidin/biotin reagent (1:1000), washed with Tris buffer, and subsequently exposed to DAB as the chromogen. All sections underwent the same processing time in bottomless wells, followed by dehydration through graded alcohols, clearing in xylene, and finally, mounted using Permount. To visualize the overall tissue structure, adjacent serial sections were stained with cresyl violet or neutral red.

For the paraffin‐embedded sections, a similar protocol was employed, as previously described.^33^, ^34^, ^35^, ^36^ In brief, 10 *µ*m paraffin‐embedded tissue sections were deparaffinized in xylene and rehydrated through a series of decreasing alcohol concentrations (100%, 95%, 80%, and 70%), followed by rinsing in distilled water. Antigen retrieval was performed by heating the sections in citrate buffer (pH 6.0) at 95°C, allowing them to cool before rinsing in PBST. Blocking was carried out using 3% BSA, and then the sections were incubated with primary antibodies overnight at 4°C. After incubation with primary antibodies (same as above), the slides were rinsed in PBST and subjected to species‐appropriate secondary antibodies for 2 h at room temperature, following the details provided above. The sections were washed and taken through the avidin/biotin amplification system, followed by DAB staining, as above. Like the free‐floating sections, the paraffin sections underwent dehydration, clearing, and mounting steps.

Positive and negative controls were employed for both IgG4 and HCMV detection. The primary IgG4 antibody (ab109493) underwent incubation with an IgG4 Peptide (#DF12638‐BP, Affinity Biosciences). Following incubation, the antibody/peptide conjugate was subjected to either free‐floating or paraffin‐embedded histochemical procedures, as previously outlined. Notably, incubation with the IgG4 antibody/peptide conjugate resulted in the complete elimination of IgG4 immunoreactivity (Figure S2).

For HCMV, specimens known to be HCMV positive (lung) and negative (myometrium) were acquired (Newcomer Supply, Part # 3240A) and subsequently incubated with anti‐HCMV antibodies as described above. As expected, the HCMV(+) lung sample exhibited reactivity, while the HCMV(−) myometrium sample showed no such reactivity (Figure S3).

Imaging of the immunostained tissue sections, stained with DAB, was performed using Nikon Eclipse Ti2 confocal and Olympus IX70 microscopes equipped with bright field and epifluorescence illumination. The results were captured using an Olympus DP‐71 color digital camera or, for confocal microscopy, a Nikon A1/A1R system.

### 2.5 Association testing between CD83(+) microglia and IHC features

Statistical analysis was performed using R Statistical Software^37^ (version 4.1.0; R Foundation for Statistical Computing, Vienna, Austria). Association testing between CD83(+) microglia and HCMV IE / HCMV Late / IgG4 Antigen immunoreactivity was performed using a two‐tailed Fisher's exact test implemented in the Fisher test function in R.

### 2.6 RNA in‐situ hybridization based validation of HCMV detection

As previously performed in our laboratory,^33^, ^38^, ^39^ we utilized an RNA in‐situ hybridization (ISH) approach for HCMV detection on formalin‐fixed, paraffin‐embedded (FFPE) human autopsy‐derived tissues, including samples from the medial temporal gyrus (Banner cohort), TC (Banner cohort), and PFC (ROSMAP cohort), using the RNAscope Multiplex Fluorescent Assay kit (Advanced Cell Diagnostics, Inc.) (Figure S4). Following the RNAscope user manual for tissue‐specific instructions, brain tissue sections were fixed, embedded in paraffin, and sectioned at 5 *µ*m thickness before being mounted on glass slides. The slides underwent deparaffinization using xylene, followed by a series of ethanol washes to rehydrate the tissue, followed by protease treatment to unmask the target RNA. Probes specific to HCMV RNA (HHV5‐IE and HHV5‐pp65) were hybridized to the tissue sections alongside appropriate control probes (positive control probe [*PPIB*] to verify RNA integrity in the human tissue samples and a negative control probe [*dapB*] to confirm the specificity of the hybridization). Detection of hybridized probes was achieved using fluorescently labeled oligonucleotide probes, which resulted in the visualization of HCMV RNA as distinct red fluorescent puncta. Nuclei were counterstained with 4′,6‐diamidino‐2‐phenylindole (DAPI), providing a blue background for clearer cellular localization of the signals. The assay was carried out using the RNAscope Multiplex Fluorescent Reagent Kit (Advanced Cell Diagnostics, Inc.) in strict adherence to the manufacturer's instructions. Slides were mounted with an antifade mounting medium and imaged using a high‐resolution confocal microscope (Nikon Metrology).

### 2.7 Cerebrospinal fluid IgG4 antiviral antibody repertoire analysis

We applied a modified version of a previously described Serum Epitope Repertoire Analysis (SERA), an assay based on a random bacterial display peptide library coupled with next‐generation sequencing (NGS), to power the development of Protein‐based Immunome Wide Association Study (PIWAS).^40^ PIWAS uses proteome‐based signals to discover candidate antibody‐antigen epitopes that are significantly elevated in a subset of cases compared to controls (in our study, CD83(+) AD vs. CD83(−) AD subjects).

Cerebrospinal fluid (CSF) samples were screened as described in,^41^ with the following modifications introduced for IgG4‐specific profiling: 100 *µ*L of CSF (1:5 dilution final) was used in the assay and incubated with the library overnight (16–18 h) on an orbital shaker at 4C. For antibody capture, a biotin‐coupled anti‐human IgG4 secondary antibody (Mouse Anti‐Human IgG4 Fc‐BIOT, Cat. No. 9200‐08, Southern Biotech) was used at a 1:100 dilution. After centrifugation and washing, cells were resuspended in 900 *µ*L of 1 × PBS plus 100 *µ*L of Dynabeads MyOne streptavidin T1‐coated magnetic beads (Thermo Fisher Scientific; catalog no. 65601).

Detected *k*‐mers for each sample were initially mapped to an expansive set of 1045 viral proteomes known or suspected of infecting humans, collectively representing 5979 individual viral protein sequences (Table S2) obtained from UniProt.^42^ The *k‐*mers were mapped to these proteomes largely according to the procedure described in the PIWAS method,^40^ with the exception that raw *k‐*mer log‐enrichment values were used without normalization to a large control cohort, due to sample restrictions. Briefly, for each sample, 5‐mer and 6‐mer counts are calculated as the number of times they appear in the unique 12‐mer reads from SERA. Expected counts are calculated based on the individual amino acid frequencies in the sample. The enrichment is calculated as the number of observed counts divided by the expected counts for that 5‐mer or 6‐mer and the log is taken. These 5‐mer and 6‐mer log‐enrichments are summed at each position and averaged over a 5 amino acid sliding window to yield a value for each residue for each sample. Finally, to summarize each sample's signal to each protein, the maximum of these residue level values is taken from across all residues on the protein, yielding a value for each sample to each protein. Further details can be found in the original PIWAS method.^40^

To identify whether there were IgG4 antibodies against any viral proteins detected at differing abundance between CD83(+) AD subjects compared with CD83(−) AD subjects, we implemented an outlier sum approach as previously described by Tibshirani and Hastie.^43^ Group level differences in OS statistics were estimated through comparison with a viral protein‐specific null distribution generated via 10,000 permutations to estimate a *Z*‐score and *p*‐value for each viral protein. *P*‐values were adjusted using the Benjamini–Hochberg procedure.^44^

### 2.8 HCMV‐focused IgG4 epitope‐level analysis

Normalized *k*‐mer (5‐mer and 6‐mer) enrichment scores were calculated for each *k*‐mer based on the number of 12‐mer reads, as previously described for the PIWAS method.^40^ First, the 12‐mers were decomposed into both 5‐mers and 6‐mers via a sliding window. We then calculated a normalized enrichment score for each *k*‐mer by taking the number of sequencing reads that contain the *k*‐mer sequence and dividing it by a proportionality constant for that *k*‐mer. The proportionality constant for each *k*‐mer is determined by taking the total number of 12‐mer reads in a sample multiplied by the product of the amino acid frequencies of each amino acid present in the *k*‐mer.

For each sample and a given amino acid sequence from a protein of interest, we next tiled 5‐mers or 6‐mers across the length of the protein. At each amino acid position, we took the sum of all 5‐mer or 6‐mer enrichment scores overlapping that position.

Next, we permuted the amino acid sequence in 12‐amino acid wide windows across the entire length of the protein and retiled the permuted area. We performed 1000 permutations for each protein and calculated a *Z*‐score for each amino acid position using the permutation scores as the null distribution for both 5‐mer and 6‐mer tiles. We choose to permute the amino acid sequence in 12‐amino acid wide windows, rather than the entire sequence to (1) account for localized repetitive sequences and (2) mimic the 12‐amino acid long display peptides. Finally, the higher *Z*‐score for each amino‐acid position between the 5‐mer or the 6‐mer was chosen as the *Z*‐score representing the enrichment for that amino acid within the protein for that sample.

Epitopes were identified via peak calling. For a given sample and protein, we plotted the amino acid position along the x‐axis and the *Z*‐score along the y‐axis. We defined an epitope as a local maxima with a height of at least 7 (reflective of the 97.5th percentile of peak heights) and a width of 6–15 amino acids (reflecting the typical epitope size recognized by antibodies).

After identifying peaks across all samples, we next clustered peaks into distinct epitope clusters. We defined a distinct epitope cluster as peaks within 20 amino acids of each other. For a cluster to be considered a distinct epitope, we required the peak to be found in at least three of the samples. Clustering was performed using complete‐linkage clustering.

To compare epitopes, we calculated the area under the curve (AUC) for each peak in the cluster by using the trapezoidal rule, as implemented by Python's sklearn module^45^ for estimating the boundaries of the peak. For samples that were negative for the epitope (no peak), we assumed the boundaries (the lower and upper end of all samples that were positive for the epitope) to calculate its AUC value.

For each epitope, we calculated the OS as described by Tibshirani and Hastie.^43^ Briefly, for each epitope, the AUC is normalized by dividing the difference of the AUC and the median epitope AUC in control samples by the median absolute deviation in control samples (in instances where the median absolute deviation was 0, we forewent dividing the difference). Next, the interquartile range (IQR) is calculated as the difference between the 75th and 25th percentile AUC value of all samples (case and control) and values in the case set greater than the 75th percentile + IQR of all samples are considered outliers. All case outliers are then summed together to get the outlier sum statistic.

After calculating OS for each sample, we performed 100,000 permutations of the case/control labels to use as the null distribution to calculate a *Z*‐score and *p*‐value. *P*‐values were corrected for multiple hypotheses using the Benjamini–Hochberg procedure^44^ (Table S3).

### 2.9 HCMV infection of cerebral organoid cells

HCMV was prepared as previously reported.^46^ Cerebral organoids were differentiated using previously reported protocols.^47^, ^48^, ^49^ Viral infections and flow cytometry (for detection of viral abundance, cell death, and AD‐associated neuropathological molecular features) experiments were optimized from our previous protocols.^50^ Briefly, dissociated cerebral organoid cells were plated 24‐h before infection at 250,000 cells/well on Matrigel‐coated plates. Cells were first washed with 1.5 mL of 1 × Dulbecco's PBS (DPBS) and inoculated for 2 h in HCMV, 1 × DPBS, and 0.5% fetal bovine serum (FBS). Following inoculation, cells were washed with 1.5 mL of 1 × DPBS and incubated in cerebral organoid differentiation media for 48 h before the flow cytometry experiments. To increase reproducibility across conditions and lab personnel, data analyses for the flow cytometry results were performed using a machine learning based gating approach on raw intensity values.^44^

## 3 RESULTS

### 3.1 CD83(+) microglia associated with IgG4 immunoreactivity in the TC

**FIGURE 1:**
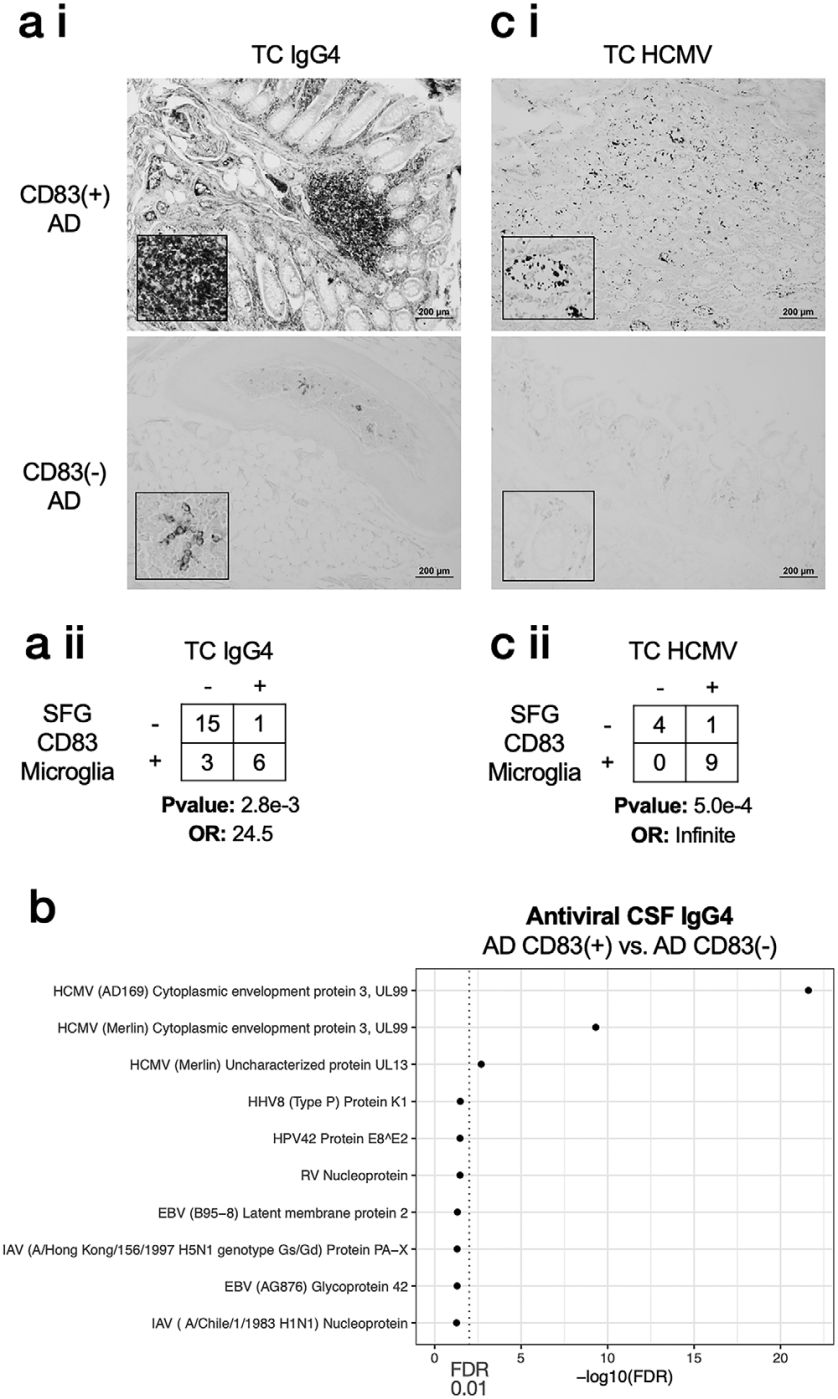
CD83(+) microglia are associated with IgG4 and HCMV in the TC. (A i–ii) Significant association between CD83(+) microglia and IgG4 immunoreactivity in TC. (B) CSF based antiviral IgG4 antibody repertoire analysis highlights several HCMV antigens at increased abundance among AD subjects with CD83(+) microglia. (C i–ii) The significant association between CD83(+) microglia and HCMV immunoreactivity in TC. All pairwise statistics were calculated using Fisher's exact test. Representative photomicrographs are shown. AD, Alzheimer's disease; CSF, cerebrospinal fluid; FDR, false discovery rate; HCMV, human cytomegalovirus; IgG4, immunoglobulin G4; TC, transverse colon

To further validate our observation of increased IGHG4 (and thus IgG4) in the TC of subjects with CD83(+) microglia, we performed IHC on TC sections from 25 subjects within the Banner cohort reported by^4^ (Figure 1A i, Figure S5) and observed a statistically significant enrichment between the presence of CD83(+) microglia in the SFG and IgG4 immunoreactivity in the TC (Figure 1A ii, *p*‐value: 2.8e‐3, odds ratio: 24.5, Fisher's exact test).

### 3.2 Anti‐HCMV IgG4 antibodies enriched in CSF of AD subjects with CD83(+) microglia

An increased abundance of IgG4 in the TC could reflect a nonspecific increase in general IgG4 antibody production or might indicate antibodies generated against specific antigens. We thus generated IgG4‐specific antiviral antibody profiles from 57 *post mortem* CSF samples from within the Banner cohort (CD83(+) microglia AD *n* = 27, CD83(−) microglia AD *n* = 30) using an epitope repertoire analysis approach.^51^, ^52^ These data allow the identification of specific antigens that react to the increased levels of IgG4. Using an outlier sum statistical approach,^40^ we observed a significantly increased abundance of IgG4 against several HCMV antigens (Figure 1B, Table S2), in particular the UL99 tegument protein also referred to as Cytoplasmic Envelopment Protein 3 (CEP3), critical for the envelopment of HCMV virions within the cytoplasm of infected cells^53^ and thus essential for viral replication. In addition, we saw an enrichment for IgG4 antibodies against UL13, capable of directly increasing cellular respiration during infection by targeting mitochondrial cristae architecture.^54^ We then performed an epitope‐level analysis focused on HCMV and identified 24 unique epitopes that were over‐represented among the IgG4 repertoires of CD83(+) microglia AD subjects (FDR < 0.05, Table S3), with the “QLRHALELQ” motif contained within the UL35 protein emerging as the most strongly enriched (*Z*‐score: 7.5, FDR 4.1e‐10). Production of UL35 by HCMV inhibits type I interferon activation via downregulation of TANK‐binding kinase 1 signaling, thus supporting viral escape of host immune responses.^55^

### 3.3 Increased HCMV in the TC of AD subjects with CD83(+) microglia

We performed IHC using an Anti‐HCMV Late antigen antibody^56^ and also an Immediate Early (IE) antibody which localizes to the nucleus of infected cells.^57^ We confirmed HCMV positivity in all nine CD83(+) TC samples evaluated, and in one CD83(−) TC sample indicating a strong positive association between HCMV within the TC and CD83(+) microglia within the SFG (Figure 1C, *p*‐value: 5.0e‐4, odds ratio: Infinite, Fisher's exact test, Figure S5).

### 3.4 Increased HCMV and IgG4 in the SFG and vagus nerve of AD subjects with CD83(+) microglia

**FIGURE 2:**
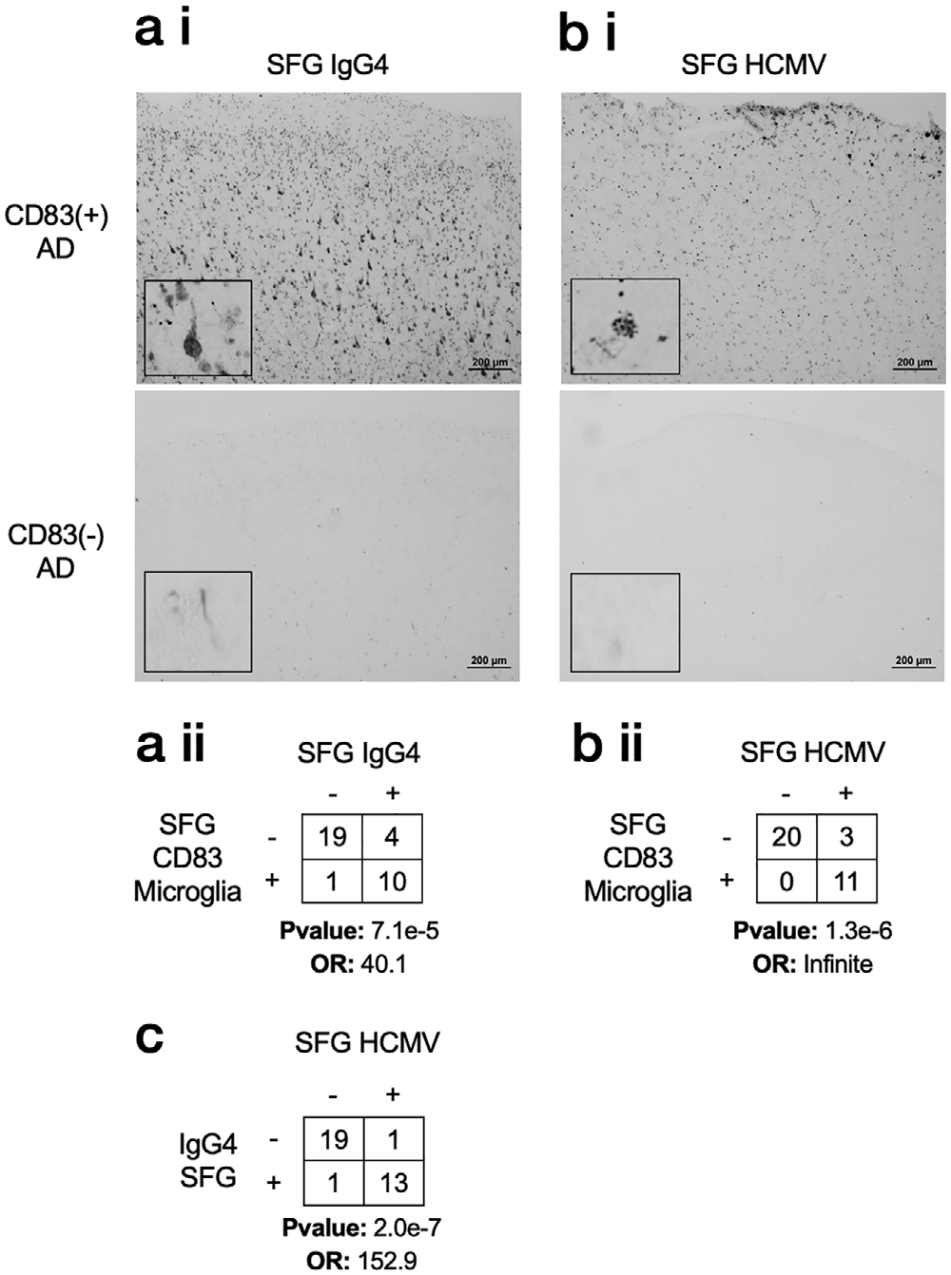
CD83(+) microglia are associated with IgG4 and HCMV presence in the SFG. (A i–ii) The significant association between CD83(+) microglia and IgG4 immunoreactivity in SFG. (B i‐ii) The significant association between CD83(+) microglia and HCMV immunoreactivity in SFG. (C) Significant association between IgG4 and HCMV immunoreactivity in SFG. All pairwise statistics were calculated using Fisher's exact test. Representative photomicrographs shown. AD, Alzheimer's disease; HCMV, human cytomegalovirus; IgG4, immunoglobulin G4; OR, odds ratio; SFG, superior frontal gyrus

Given the increased abundance of IgG4 immunoglobulins among CD83(+) AD subjects, including evidence suggestive of anti‐HCMV specificity and HCMV in the TC, we hypothesized that either IgG4 or HCMV itself may be directly inducing CD83(+) microglia in the SFG. IHC demonstrated a significant over‐representation of IgG4 positivity in the SFG of CD83(+) microglia subjects (Figure 2A, *p*‐value: 7.1e‐5, odds ratio: 40.1, Fisher's exact test, Figure S6). Of the 11 CD83(+) SFG samples examined, 10 were definitively immunoreactive for IgG4 and 1 was weakly immunoreactive (though classified as negative for statistical evaluation).

**FIGURE 3:**
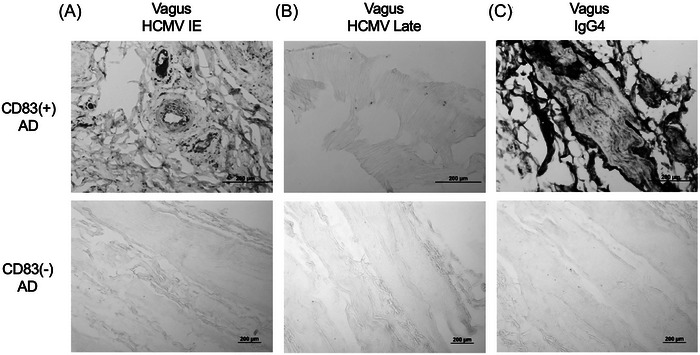
IgG4 and HCMV presence in the vagus nerve of AD subjects with CD83(+) microglia. (A–B) HCMV and (B) IgG4 immunoreactivity in the vagus nerve of AD subjects with and without CD83(+) microglia. Representative photomicrographs shown. AD, Alzheimer's disease; HCMV, human cytomegalovirus; IgG4, immunoglobulin G4.

We then performed HCMV IHC on SFG brain tissue sections from 34 subjects (CD83(+) *n* = 11, CD83(−) *n* = 23) and observed HCMV immunoreactivity in 14 samples (CD83(+) *n* = 11, CD83(−) *n* = 3), with every CD83(+) sample demonstrating the presence of HCMV (Figure 2B, *p*‐value: 1.3e‐6, odds ratio: Infinite, Fishers exact test, Figure S6). Within the SFG, HCMV was also significantly associated with IgG4 (Figure 2C, *p*‐value: 2.0e‐7, odds ratio: 152.9, Fishers exact test). We hypothesized that the vagus nerve might represent a potential route of passage for viral particles between the TC and the brain,^58^ and so examined vagal tissue sections (collected adjacent to the carotid body) for five CD83(+) and three CD83(−) subjects (Figure 3, Figure S7). All five CD83(+) sections demonstrated immunoreactivity to IgG4, HCMV IE, and Late antigens, with two CD83(‐) sections demonstrating immunoreactivity to HCMV IE and no sections immunoreactive to the HCMV Late antigen or IgG4. Overall, the histochemical staining patterns observed in TC, SFG, and vagus nerve of CD83(+) subjects are consistent with active HCMV infection. Taken together, these results indicate a multiorgan presence of IgG4 and HCMV in subjects with CD83(+) microglia within the SFG.

### 3.5 Replication of cortical HCMV association with CD83(+) microglia in an independent AD cohort

We wondered whether HCMV might also be detectable in association with CD83(+) microglia within brain tissue sections from an independent cohort of subjects with AD. Recently, Fujita et al.^23^ and Mathys et al.^24^ published analyses based on snRNA‐seq from 433 and 427 PFC samples, respectively, from subjects included within the ROSMAP cohort.^25^ As reported in^4^ we performed DAseq using the same workflow we had applied to the Banner SFG snRNA‐seq to identify CD83(+) AD subjects. We then obtained PFC tissue sections from 13 CD83(+) AD and 14 CD83(−) AD subjects and performed anti‐IE1 HCMV IHC. We observed HCMV immunoreactivity in 13 samples (CD83(+) *n* = 10, CD83(−) *n* = 3), notably in association with dense core plaques and CD83(+) microglia (Figure S1A), representing a significant overlap between HCMV immunoreactivity and CD83(+) status (*p*‐value: 7.0e‐3, odds ratio: 10.8, Fishers exact test, Figure S1B).

### 3.6 Validation of IHC detection of HCMV

We verified the specificity of our HCMV IHC findings through the inclusion of known HCMV positive (lung) and negative (myometrium) samples which were correctly classified as positive and negative respectively (Figure S3). We performed further validations using an orthogonal RNA ISH approach in brain and TC tissue samples from subjects in the Banner and ROSMAP cohort (Figure S4). These studies yielded results consistent with those expected from our histochemical findings of HCMV presence.

### 3.7 HCMV infection of human cerebral organoids accelerates Aβ42 and pTau‐212 production, and induces neuronal death

**FIGURE 4:**
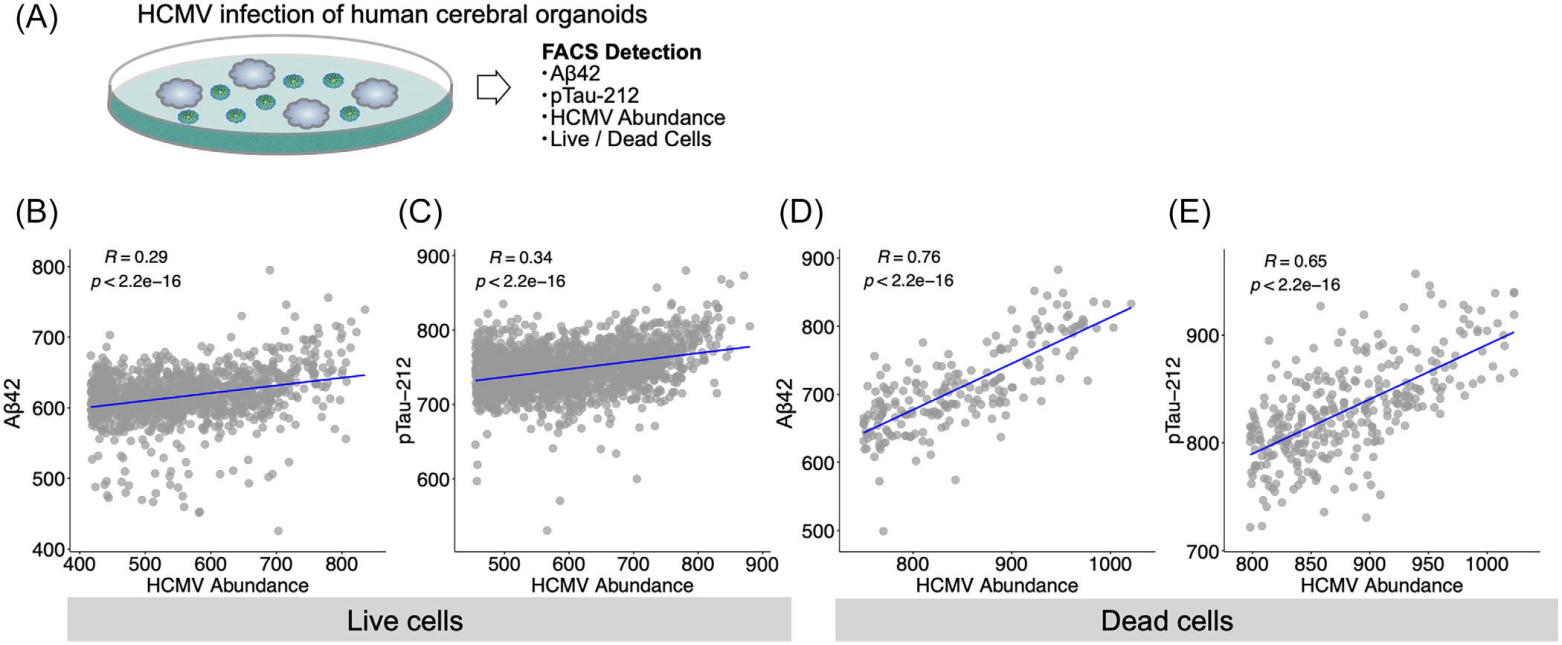
HCMV infection of human cerebral organoids accelerates AD neuropathological features. (A) HCMV infection of human cerebral organoids (MOI = 2) demonstrates significant correlations between Aβ42 and pTau‐212, and HCMV abundance in (B–C) live and (D–E) dead cells. Representative photomicrographs shown. Aβ42, amyloid beta‐42; AD, Alzheimer's disease; CSF, cerebrospinal fluid; HCMV, human cytomegalovirus; MOI, multiplicity of infection; *p*, *p*‐value; pTau‐212, phosphorylated Tau‐Thr212; OR, odds ratio; *r*, Pearson's correlation.

Human cerebral organoids comprise a complex mixture of neurons and glial cells and enable the study of brain structure and function in a manner that reflects the strengths of human and animal model systems. We therefore performed live HCMV infection of human cerebral organoids (multiplicity of infection [MOI] = 2) followed by flow cytometry based detection of viral abundance, cell death markers, and AD‐associated neuropathological molecular features amyloid beta‐42 (Aβ42) and phosphorylated Tau‐Thr212 (pTau‐212) (Figure 4A) using our previous protocols.^59^ We observed high, positive correlations between the abundance of HCMV, and both Aβ42 (Figure 4B, D) and pTau‐212 (Figure 4C, E). We observed the strongest correlation within dead cells (stained with a Zombie dye) (Pearson's *r* = 0.47–0.76 and 0.65–0.67, respectively, two replicates), with this pool of cells enriched for dead neurons. We observed comparable results at a higher MOI of 4 (Figure S8). These findings suggest the capacity of HCMV to accelerate intracellular production of pathophysiological features of AD (Aβ42 and pTau‐212) as well as inducing neuronal death.

### 3.8 HCMV infection induces expression of CD83 in C20 microglia

Given that CD83 is a known marker of activated antigen presenting cells,^60^ we hypothesized that in the CD83(+) microglia in HCMV(+) subjects may have been directly infected or have digested HCMV virions. We therefore infected immortalized C20 microglia cells with HCMV, human herpesvirus‐6A (HHV‐6A, a related double‐stranded DNA human herpesvirus), and lipopolysaccharide (LPS) and compared these with mock‐infected cells (Figure S9). We observed significant upregulation of CD83 in HCMV infected microglia (but not HHV‐6A or LPS treated) compared with mock infected cells at several MOI. These findings indicate that HCMV is sufficient to induce CD83 in microglia and that this does not appear to be a universal response to other viral infections or immune perturbations.

## 4 DISCUSSION

IgG4 is one of four main IgG immunoglobulin subclasses, and the least prevalent among healthy adults (approximately 5% of IgG) with structural differences accounting for reduced triggering of antibody‐dependent cell‐mediated phagocytosis, reduced cross‐linking of antigens and reduced complement activation.^61^ Although it is generally accepted that IgG4 exerts a comparatively anti‐inflammatory effect relative to other IgG subclasses, IgG4 is still capable of driving phagocytosis of opsonized microbial antigens^62^ and is capable of mediating complement activation at higher antigen and antibody concentrations.^63^ IgG4 is not commonly observed as part of the humoral immune response to viruses,^64^ although detectable HCMV‐specific IgG4 has been reported to occur in some individuals.^65^

Previous studies have reported CD83(+) microglia in AD^3^ and recent findings implicate CD83 as a marker of microglia engaged in myelin debris phagocytosis as well as a potential modulator of autoimmune neuroinflammation during certain disease states.^66^ Notably, HCMV UL86 peptide 981–1003 is capable of cross‐reacting with myelin oligodendrocyte glycoprotein peptide 35–55 such that HCMV infection can induce experimental autoimmune encephalomyelitis (EAE) in Lewis rats.^67^ Given that CD83(+) microglia are also reported to modulate EAE,^66^ one possibility is that while they perform a critical role in the clearance of myelin debris, this activity may also be exacerbated by microbial interactions, including via molecular mimicry mechanisms. Our observations that HCMV infection of C20 microglia is sufficient to induce CD83 is consistent (but not dispositive) with HCMV being a direct cause of the CD83(+) microglia we originally observed in‐situ within the SFG snRNA‐seq.^4^

We also note that infection of human cerebral organoids with HCMV is capable of inducing accumulation of intracellular pathophysiological features of AD (Aβ42 and pTau‐212) as well as causing neuronal death, at two different MOI. The capacity of HCMV to induce molecular precursors of AD molecular pathology in an organoid model system may also explain our previous observation of increased neurofibrillary tangle and neuritic plaque pathology in CD83(+) AD subjects compared with CD83(−) AD subjects within the ROSMAP cohort.^4^ Although we observed significant positive correlations between HCMV and Aβ42/pTau‐212 across all replicates and MOI, we did observe some variability in the magnitude of the correlations, particularly between the live populations of cells. Since these correlations are mediated primarily through neurons, this variability may be reflective of comparatively minor fluctuations in neuronal fractions within the living and dead cell populations across experiments.

In the presence of actively infected brain cells, one explanation of our observations is that both CD83(+) microglia and IgG4 indicate mechanisms to increase immunological tolerance toward HCMV, restraining a more aggressive antiviral immunity which might otherwise accentuate the neuronal loss that is characteristic of AD.^68^ An alternative is that these mechanisms reflect a viral manipulation of host biology and could thus warrant therapeutic modulation. HCMV IgG seroprevalence is common and varies by age and comorbidity, present in 79% of 85‐year‐olds.^69^ Despite this, we note that HCMV presence in the TC was not ubiquitous and was significantly associated with CD83(+) microglia and HCMV in the SFG. This observation may help reconcile how a common pathogen might contribute to a disease that most individuals do not develop. Evidence of HCMV within the vagus nerve is consistent with the transvagal passage of virions to the brain though does not preclude additional routes of infection. An alternative route of entry could be the passage of HCMV‐infected CD14(+) monocytes that migrate into the brain from the periphery^70^ and differentiate into microglia. Determination of the factors that may predispose to active (and likely chronic) HCMV infection in the TC, despite near universal exposure, is also unresolved. Potential explanations that could warrant further investigation may include HCMV strain‐specific virulence factors, microbial coinfections, interactions with a facilitative microbial dysbiosis,^71^ differing reactivation frequency,^72^ or immunosenescence.^9^ Delineation of these functional relationships and their contribution to core AD pathomechanisms will require careful follow‐up work in additional tissues and AD model systems, as well as evaluation of equivalent findings in additional brain regions and clinical cohorts.

In conclusion, we present a study aimed at contextualizing the relevance of a recently described AD‐associated CD83(+) microglia subtype. Through the integration of body tissue samples collected from the same subjects whose SFG snRNAseq led to the identification of the CD83(+) subtype, we report a series of significant associations linking CD83(+) microglia in the SFG with IgG4 and HCMV presence in the TC, anti‐HCMV IgG4 antibodies in the CSF, and both IgG4 and HCMV in the vagus nerve and SFG. HCMV histochemistry is consistent with an active HCMV infection, which may indicate an opportunity for the administration of antiviral therapy in subjects with AD and biomarker evidence of HCMV, IgG4, or CD83(+) microglia.

## CONFLICT OF INTEREST STATEMENT

S.G. has served as a consultant in the past for J&J, Diagenic, and Pfizer, and he currently consults for Cognito Therapeutics, GLG Group, SVB Securities, Guidepoint, Third Bridge, MEDACORP, Altpep, Vigil Neurosciences, and Eisai. Q.W., D.M., E.R., and B.R. are listed as co‐inventors on a patent application for an IgG4‐based peripheral biomarker for the detection of CD83(+) microglia. The other authors declare no relevant conflicts. Author disclosures are available in the Supporting Information.

## CONSENT STATEMENT

All Banner participants were volunteers in the AZSAND, a longitudinal clinicopathological study of aging, cognition, and movement in the elderly since 1996 in Sun City, Arizona. Autopsies are performed by the Banner Sun Health Research Institute Brain and Body Donation Program^5^ (BBDP; www.brainandbodydonationprogram.org). All subjects sign Institutional Review Board‐approved informed consents allowing both clinical assessments during life and several options for brain and/or bodily organ donation after death. All ROSMAP participants enrolled without known dementia and agreed to detailed clinical evaluation and brain donation at death.^25^ Both studies were approved by an Institutional Review Board of Rush University Medical Center (ROS IRB# L91020181, MAP IRB# L86121802). Both studies were conducted according to the principles expressed in the Declaration of Helsinki. Each participant signed an informed consent, Anatomic Gift Act, and an RADC Repository consent (IRB# L99032481) allowing his or her data and biospecimens to be repurposed. Details of the clinical and pathologic methods have been previously reported.^26^, ^27^, ^28^, ^29^, ^30^ Details of Banner and ROSMAP cohort participants are included here (Table S1).

## Supporting information



Supporting Information

Supporting Information

Supporting Information

Supporting Information
