# Supplementary material for: Alzheimer's disease‐associated CD83(+) microglia are linked with increased immunoglobulin G4 and human cytomegalovirus in the gut, vagal nerve, and brain
Source: Alzheimers Dement. 2024 Dec 19;21(1):e14401. doi: 10.1002/alz.14401 (PMC11772737; doi:10.1002/alz.14401)
Supplement: Supplementary file 3 — Supporting Information [file ALZ-21-e14401-s004.pdf]

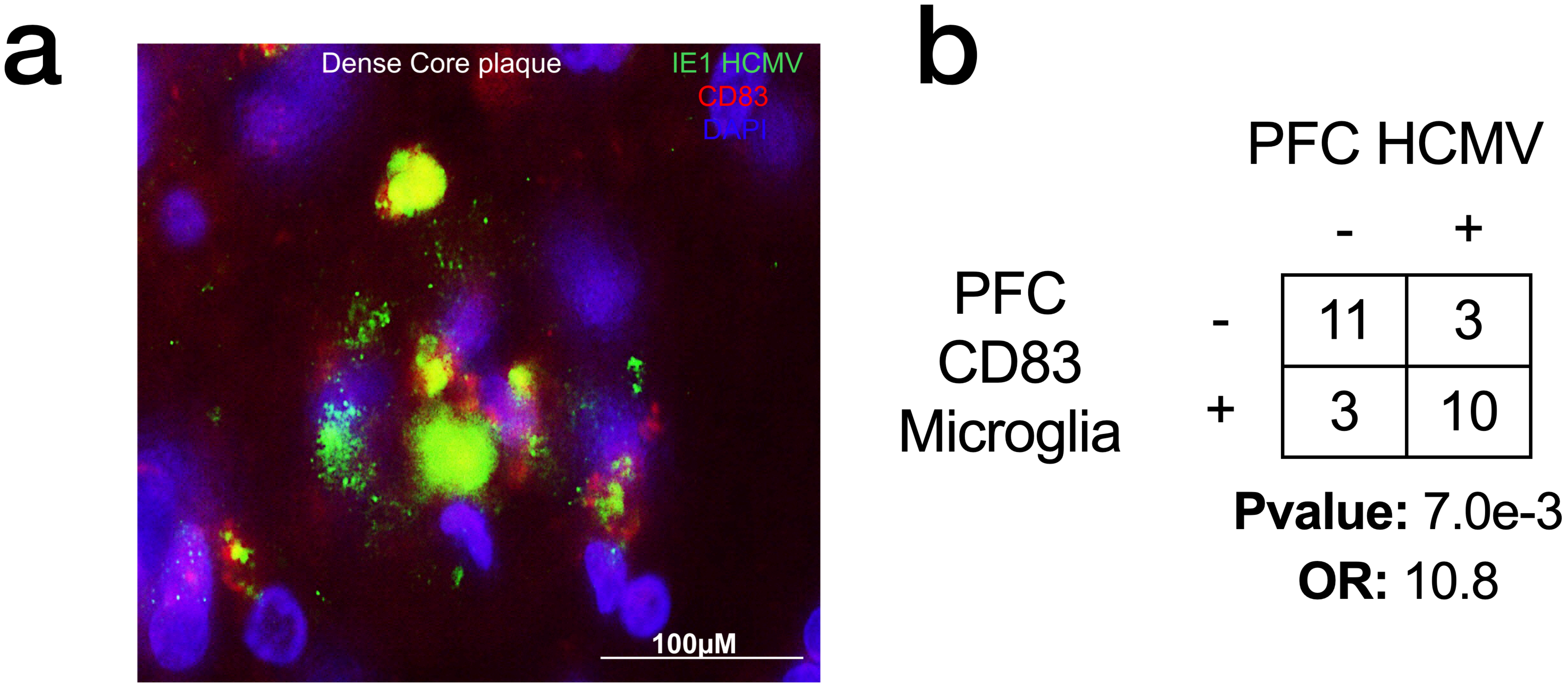

Supplementary Figure S1: Immunohistochemistry of prefrontal cortex samples from 27 AD subjects within the ROSMAP cohort (A) Representative confocal image of colocalization of CD83, DAPI and HCMV IE1 antigens around dense core amyloid plaque (B) Significant association between CD83(+) microglia and HCMV IE1 immunoreactivity in PFC within the ROSMAP cohort.

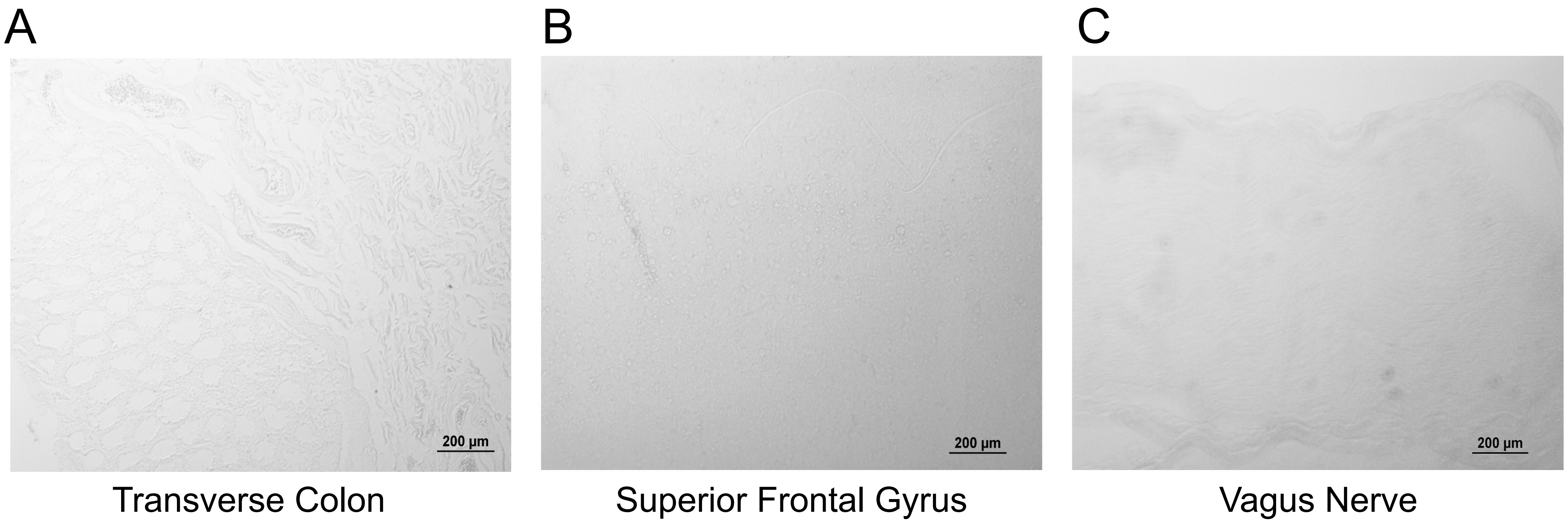

Supplementary Figure S2: Elimination of IgG4 immunoreactivity following incubation with IgG4 blocking peptide in IgG4 positive (A) Transverse Colon, (B) Superior Frontal Gyrus, and (C) Vagus Nerve samples demonstrates IgG4 detection antibody specificity.

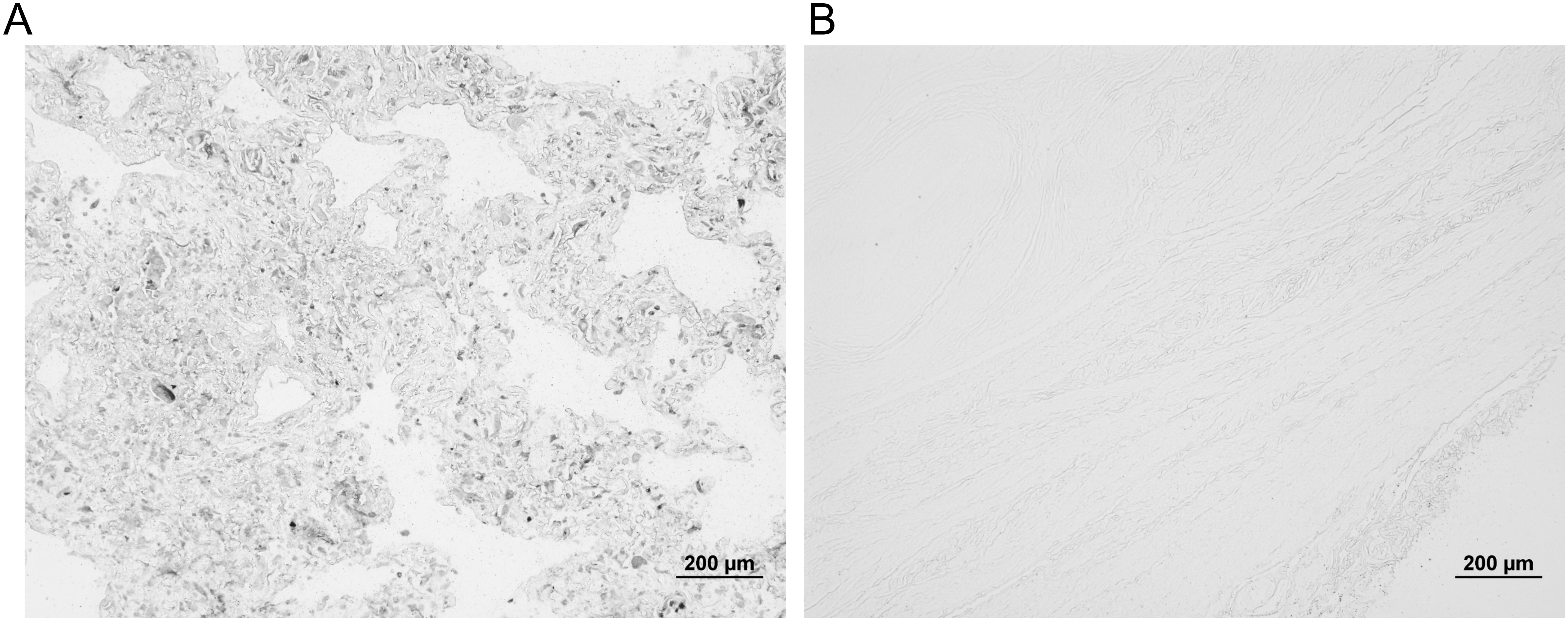

HCMV Positive Control, Lung

HCMV Negative Control, Myometrium

Supplementary Figure S3: Expected HCMV immunoreactivity in known (A) HCMV positive lung and (B) HCMV negative myometrium tissue controls demonstrates HCMV detection antibody specificity.

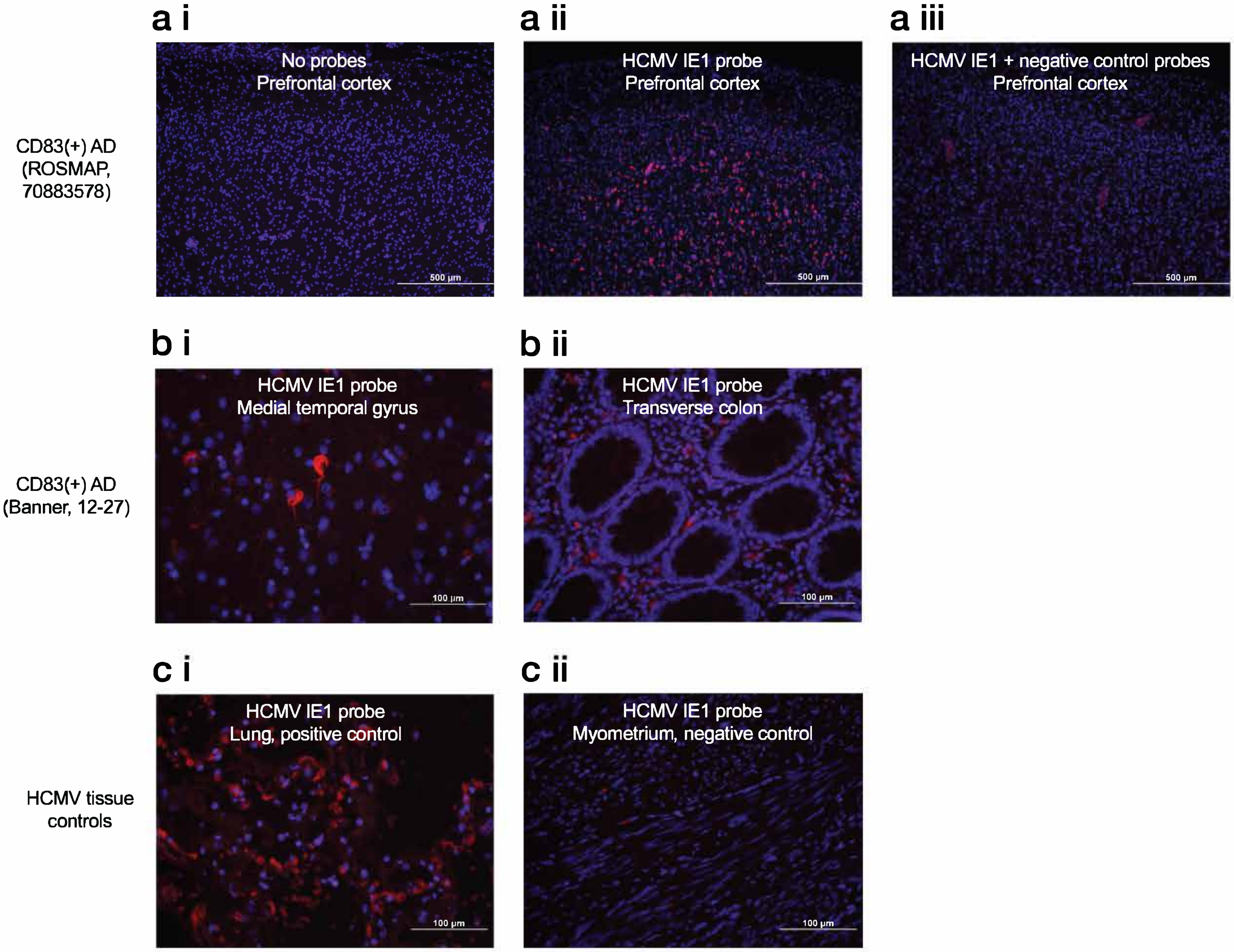

Supplementary Figure S4: RNA *in situ* hybridization validation of HCMV detection using RNAscope. (A) Detection of HCMV IE1 RNA in prefrontal cortex tissue samples from a CD83(+) AD / HCMV immunoreactive ROSMAP subject (i) with no probes added (ii) HCMV IE1 probe added, demonstrating expected positivity, (iii) which is lost with addition of negative control probe. (B) Detection of HCMV IE1 RNA in tissue samples from a CD83(+) AD / HCMV immunoreactive Banner subject demonstrating expected positivity in (i) medial temporal gyrus cortex, and (ii) transverse colon. (C) Detection of HCMV IE1 RNA in tissue samples from positive and negative HCMV control subjects with known HCMV status including (i) Lung (positive) and (ii) myometrium (negative) samples. HCMV RNA shown as distinct red fluorescent puncta. Cell nuclei counterstained with DAPI (blue).

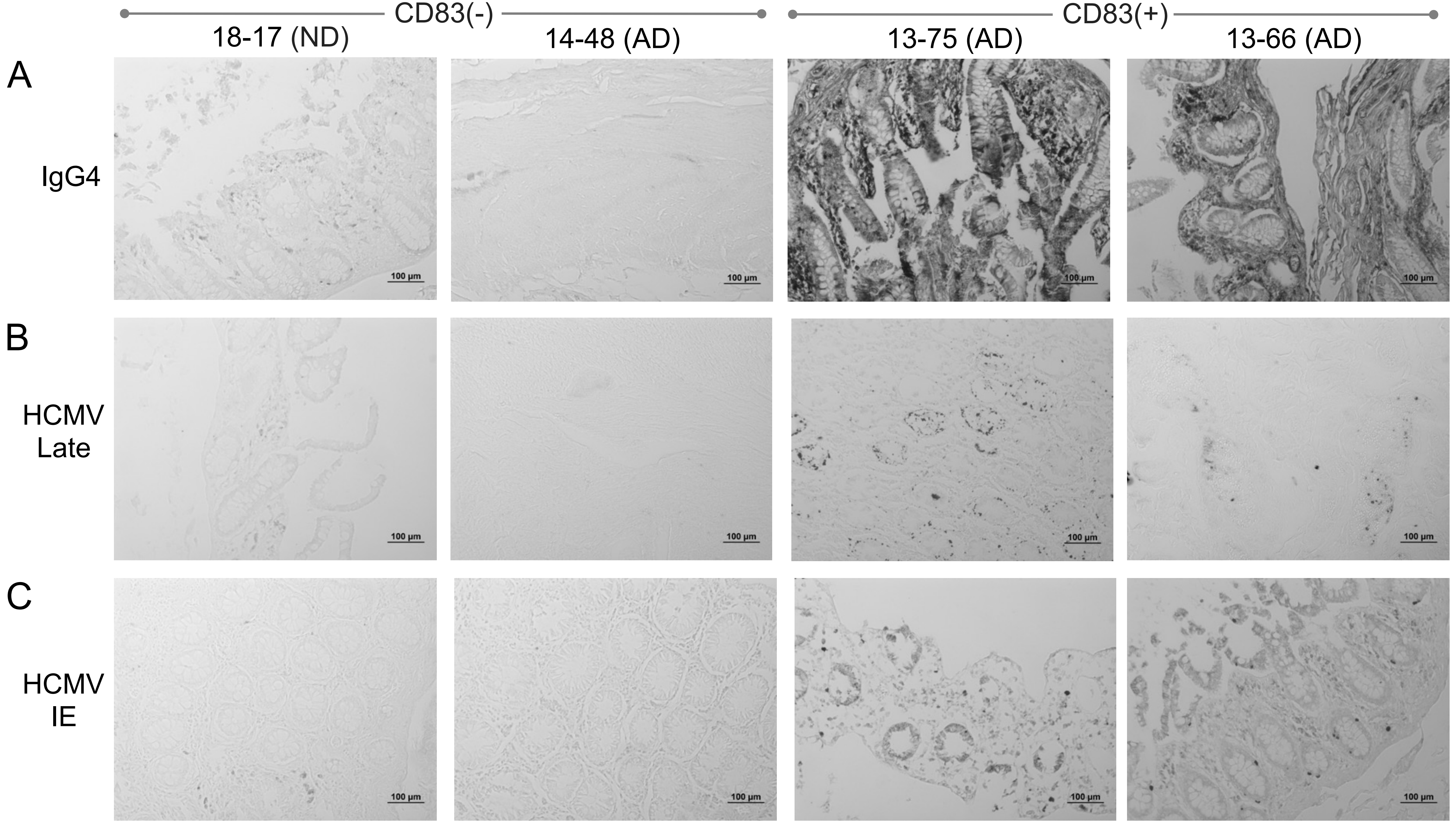

Supplementary Figure S5: Immunohistochemistry of Transverse Colon sections against (A) IgG4, (B) HCMV (Late Antigen), and (C) HCMV (Immediate Early Antigen) in representative CD83(+) and CD83(-) subjects.

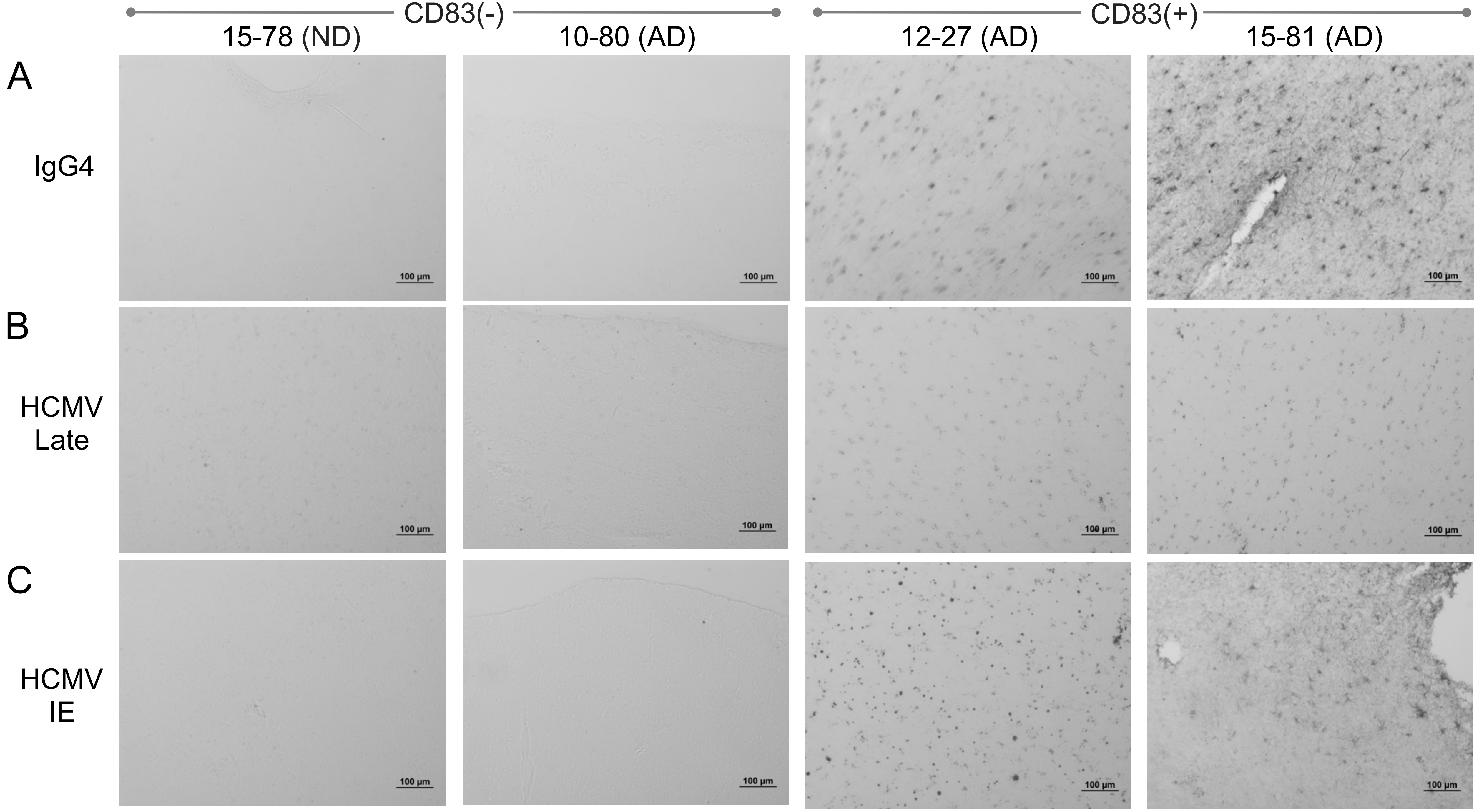

Supplementary Figure S6: Immunohistochemistry of Superior Frontal Gyrus sections against (A) IgG4, (B) HCMV (Late Antigen), and (C) HCMV (Immediate Early Antigen) in representative CD83(+) and CD83(-) subjects.

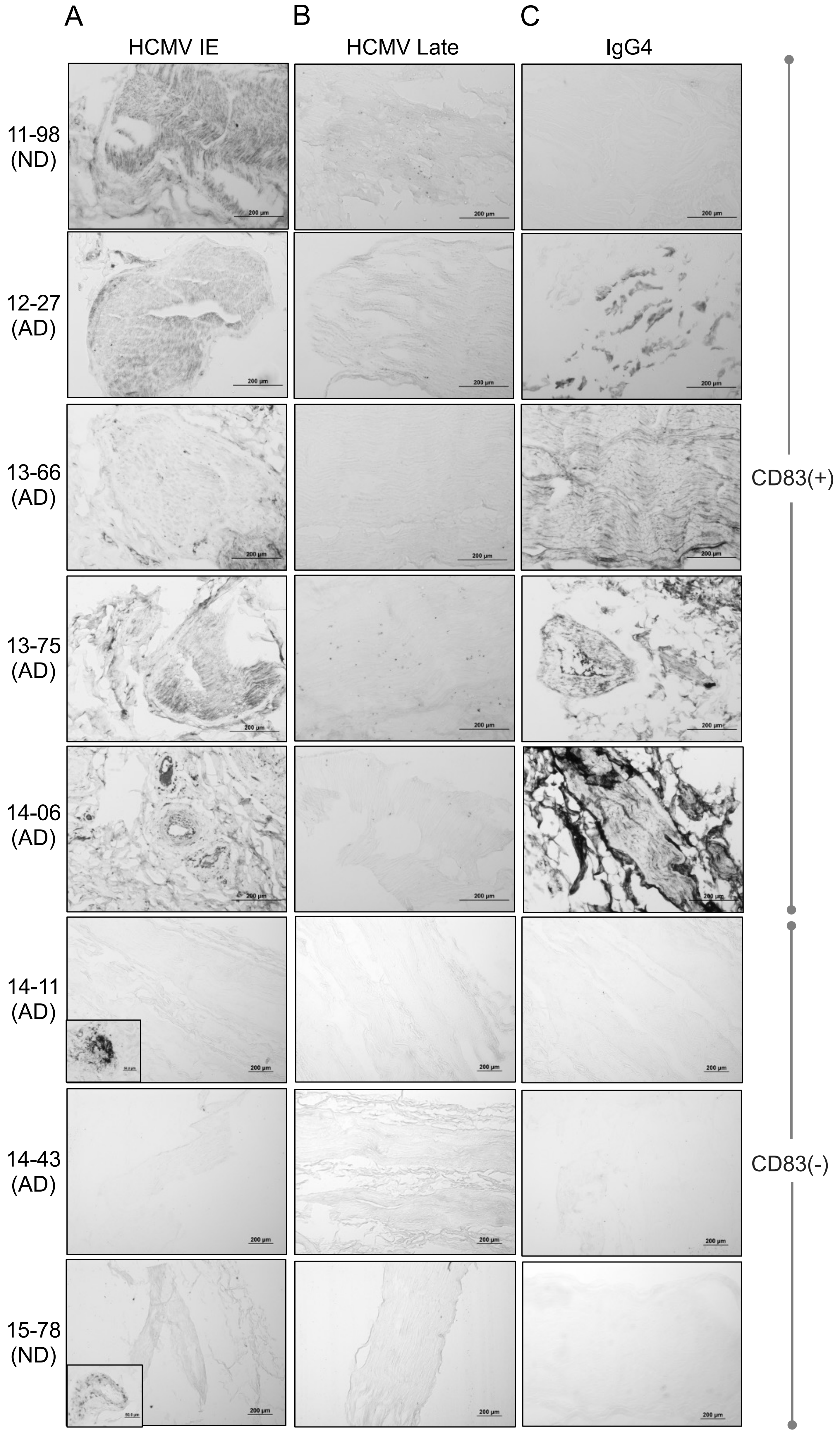

Supplementary Figure S7: Immunohistochemistry of Vagus nerve sections against (A) HCMV (Late Antigen), and (B) HCMV (Immediate Early Antigen), and (C) IgG4 in representative CD83(+) and CD83(-) subjects.

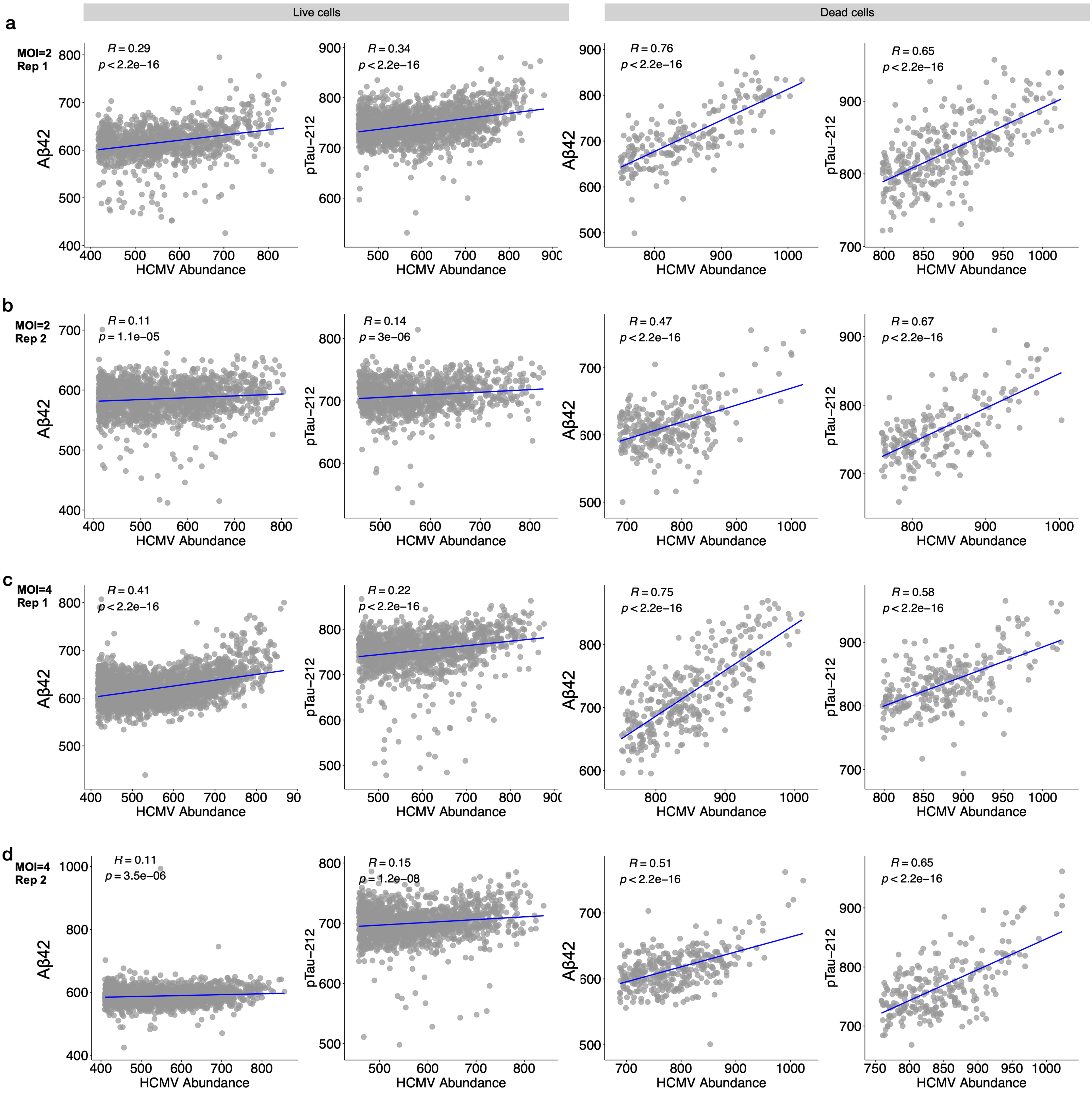

Supplementary Figure S8: Aβ42 and pTau-212 associations with HCMV abundance within infected human cerebral organoid cells. (A) MOI=2, Replicate 1, (B) MOI=2, Replicate 2, (A) MOI=4, Replicate 1, (A) MOI=4, Replicate 2. MOI: Multiplicity of Infection, p: Pvalue.

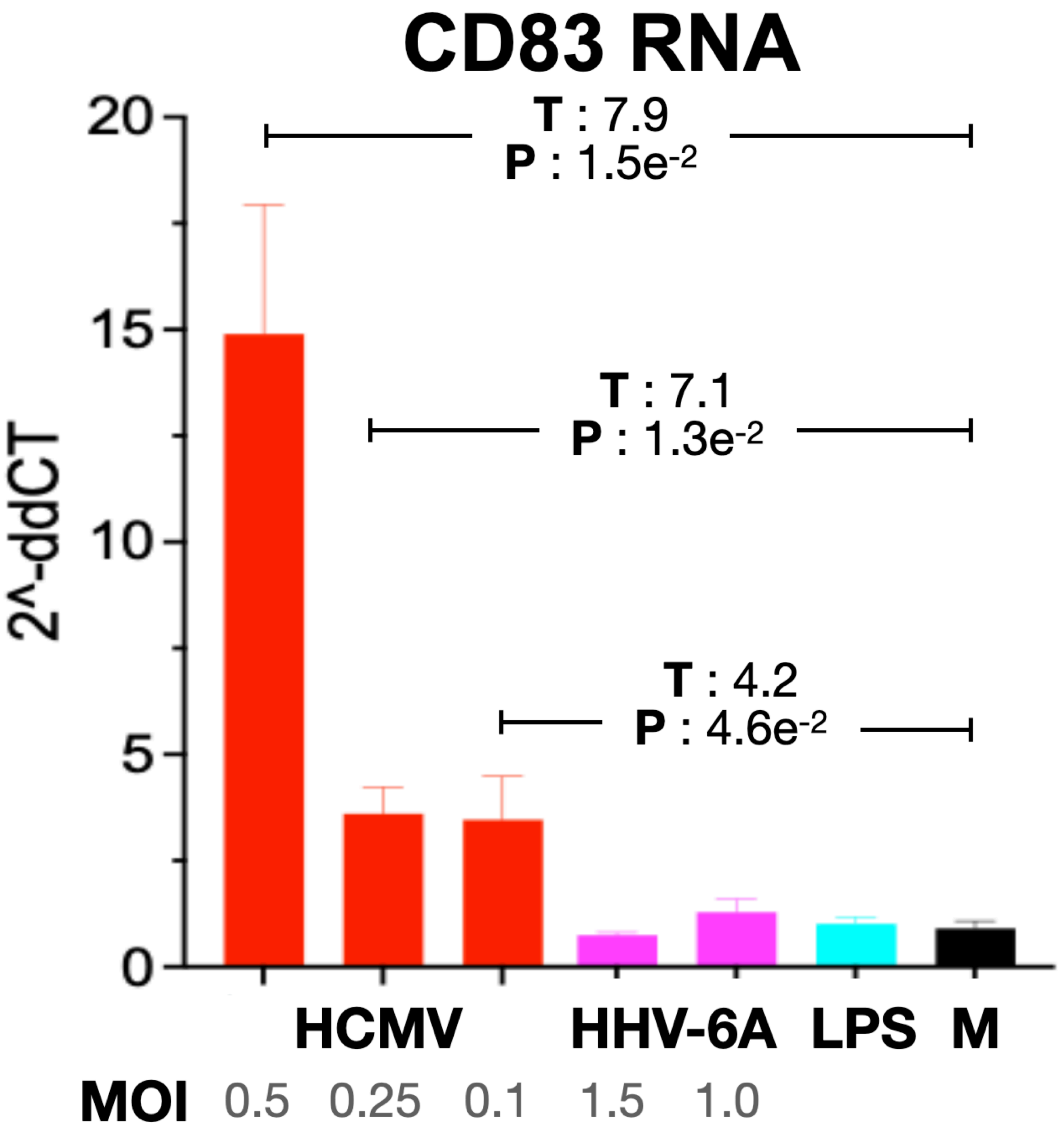

Supplementary Figure S9: Induction of CD83 expression in C20 microglia following infection with HCMV, but not HHV-6A, LPS treatment. HHV-6A: Human Herpesvirus 6A, LPS: Lipopolysaccharide, M: Mock infection, MOI: Multiplicity of Infection, p: Pvalue, T: T-statistic.
